# Salvage Robot-Assisted Minimally Invasive Esophagectomy (RAMIE) for T4b Esophageal Cancer After Definitive Chemoradiotherapy

**DOI:** 10.1245/s10434-020-09425-2

**Published:** 2020-12-19

**Authors:** I. L. Defize, S. van der Horst, M. Bülbul, N. Haj Mohammad, S. Mook, G. J. Meijer, L. A. A. Brosens, J. P. Ruurda, R. van Hillegersberg

**Affiliations:** 1 Department of Surgery, G04.228, University Medical Center Utrecht, Heidelberglaan 100, 3584 CX Utrecht, The Netherlands; 2 Department of Radiation Oncology, University Medical Center Utrecht, Utrecht, The Netherlands; 3 Department of Pulmonary Diseases, University Medical Center Utrecht, Utrecht, The Netherlands; 4 Department of Medical Oncology, University Medical Center Utrecht, Utrecht, The Netherlands; 5 Department of Pathology, University Medical Center Utrecht, Utrecht, The Netherlands

## Abstract

**Background:**

Patients  with esophageal cancer  that invades adjacent structures (cT4b) are precluded from surgery and usually treated with definitive chemoradiotherapy (dCRT). dCRT might result in sufficient downstaging to enable a radical resection, possibly improving survival. This study aimed to assess the perioperative and oncologic outcomes of a salvage robot-assisted minimally invasive esophagectomy (RAMIE) in patients with cT4b esophageal cancer after dCRT.

**Methods:**

Between June 2012 and November 2019, patients who underwent a RAMIE with a gastric conduit reconstruction after completion of dCRT for cT4b esophageal carcinoma were identified from a prospectively maintained surgical database at the University Medical Center Utrecht.

**Results:**

In total, 24 patients with a histopathologically confirmed T4b adenocarcinoma or squamous cell carcinoma of the esophagus were included. The adjacent organs involved were the tracheobronchial tree (67%), aorta (21%) or both (13%). No conversions or major intraoperative complications were observed. A radical resection was achieved in 22 patients (92%), and a pathologic complete response was observed in 13 (54%) patients. Postoperative grade 2 or higher complications occurred in 20 patients (83%). The disease-free survival at 24 months was 68% for the patients in whom a radical resection was achieved.

**Conclusion:**

In patients with cT4b esophageal cancer treated with dCRT followed by a salvage RAMIE, a radical resection rate of 92% was achieved, with acceptable complications and promising survival rates. These results demonstrate the feasibility of a curative surgical treatment for patients with initially irresectable esophageal cancer but underscore the importance of a proper preoperative patient selection.

Patients with esophageal cancer  that invades adjacent structures such as the trachea or aorta (cT4b) often are precluded from surgery and treated with definitive chemoradiotherapy (dCRT).^1^ dCRT  alone results in high locoregional failure rates  of up to 50% and low 3-year survival rates of 20% to 25%.^2^^–^7

Neoadjuvant chemoradiotherapy often results in regression of the primary tumor, enabling a radical, curative resection of initially irresectable disease.^8^^–^10

Previous reports have demonstrated favorable results in patients who underwent dCRT with subsequent surgery compared with patients who underwent dCRT alone in terms of local control as well as short- and long-term prognoses. However, these results depend largely on completeness of the resection  since the median survival of patients who  undergo an incomplete resection rarely exceeds the 6 months.^11^^–^13

Radical resection rates for salvage esophagectomies used to treat cT4b tumors vary widely, and high perioperative complication rates are observed, emphasizing the complexity of this procedure.^9^^,^^14^^–^19 Therefore, strategies to improve these surgical outcomes should be explored.

In addition to accurate assessment of tumor regression and resectability during restaging, one of the surgical strategies is robot-assisted minimally invasive esophagectomy (RAMIE). In a recently published randomized trial, RAMIE was compared with open esophagectomy for resectable esophageal cancer.^20^ The findings showed RAMIE to be superior, with fewer perioperative complications and similar oncologic outcomes.

The robot-assisted procedure offers a 10-fold magnification and a stable, three-dimensional (3D) endoscopic view of the operation field. Combined with the dexterity of the articulating instruments, a very precise dissection of the esophagus is facilitated, even in difficult areas along the tissue planes of the surrounding structures.^21^^–^23 These features may well improve the radical resection rate for downstaged cT4b tumors, which is pivotal in achieving good oncologic outcomes.

Therefore, the current study aimed to assess the perioperative and oncologic outcomes of a salvage RAMIE after dCRT in T4b esophageal cancer patients.

## Methods

### Data Collection

Data were collected from a prospectively maintained surgical database at the University Medical Center Utrecht. In accordance with the Institutional Review Board, the informed consent requirement was waived for this study.

### Study Population

Patients with a clinical and histopathologically confirmed cT4b adenocarcinoma or squamous cell carcinoma for whom curative treatment was considered possible by a multidisciplinary team at diagnosis were eligible for inclusion in the study. Curative treatment consisted of definitive chemoradiotherapy followed by a robot-assisted minimally invasive esophagectomy that involved a robot-assisted thoracoscopic phase and a laparoscopic abdominal phase.

### Clinical Staging and Restaging

The standard diagnostic workup for clinical staging consisted of an endoscopy with biopsies, an endoscopic ultrasound (EUS), and/or a positron emission tomography (PET) and computed tomography (CT) of the abdomen, thorax, and neck. Tracheobronchial tree invasion was assessed by endobronchial ultrasound (EBUS). Involvement of the aorta was defined as encasement of more than 90° on diagnostic CT confirmed by EUS provided no stenosis was impeding the EUS. Patients were restaged with the same diagnostic methods 6 to 8 weeks after completion of dCRT. Patients without signs of distant metastasis proceeded to surgery. Staging and restaging were performed according to the seventh edition of the American Joint Committee on Cancer (AJCC).^24^

### Definitive Chemoradiotherapy

Patients were treated with dCRT according to an extended ChemoRadiotherapy for Oesophageal cancer followed by Surgery Study (CROSS) regimen involving six weekly cycles of intravenous carboplatin [area under the curve (AUC), 2 mg/ml/min] and paclitaxel (50 mg/m^2^) with concurrent radiation therapy (50.4 Gy in 28 fractions of 1.8 Gy). Volumetric arc therapy based on 3D planning of CT was used for treatment planning and delivery.^25^

### Surgical Procedure

All patients who proceeded to surgery underwent a salvage RAMIE with a two-field lymphadenectomy as previously described.^26^ For the thoracic part of the procedure, the patient was positioned in the left lateral decubitus position tilted toward the prone position. Thoracoscopic mobilization of the esophagus was combined with a thoracic lymphadenectomy, which included the right (R) and left (L) paratracheal lymph nodes (station 2 R and L), the tracheobronchial lymph nodes (station 4 R and L), the paraaortic nodes (station 6), the subcarinal nodes (station 7), the peri-esophageal nodes (station 8), and the pulmonary ligament nodes (station 9), and resection of the thoracic duct compartment containing the thoracic duct and the thoracic duct lymph nodes.^27^

Before the abdominal phase, the patient was placed in the supine position. The laparoscopic gastric mobilization and abdominal lymphadenectomy included the right and left cardiac lymph nodes (stations 1 and 2), the lesser omental lymph nodes (station 3), the left gastric artery nodes (station 7), the celiac artery nodes (station 9), and the root portion of the hepatic and splenic artery (stations 8 and 11). After gastric conduit formation, an intrathoracic or cervical handsewn end-to-side esophagogastrostomy was constructed depending on the location of the tumor.

### Pathologic Assessment

A standard protocol was used to evaluate the resection specimen.^26^ The pathology report included tumor type, diameter, depth of invasion into the esophageal wall, margin status (R0 [margins not involved] and R1 [microscopic tumor residual in resection margin]), tumor regression score according to Mandard, and lymph node status.^28^ Pathologic assessment was performed according to the College of American Pathologists.^29^

### Postoperative Management

Enteral tube feeding by a feeding jejunostomy was started on postoperative day (POD) 1. Oral intake was started between PODs 4 and 7 provided no signs of anastomotic leakage were observed.

### Postoperative Complications

Postoperative complications were prospectively registered during a weekly consensus meeting. Complications were defined and severity was scored according to the Esophagectomy Complications Consensus Group and Clavien-Dindo classification.^30^,^31^

### Follow-up 

Follow-up was performed according to the Dutch guidelines including outpatient clinic visits at 3, 6, 9, and 12 months during the first postoperative year, then half-yearly visits during the second year and annual visits during the third, fourth, and fifth years. If the patient experienced symptoms of tumor recurrence, additional diagnostic tests were performed.

### Statistical Analysis

Patient and tumor characteristics were described as counts with percentages, means, with standard deviations or as medians with ranges where appropriate. Overall survival (OS) and disease-free survival (DFS) were assessed according to the Kaplan–Meier method. The OS was calculated from the start of dCRT to death. For the patients who underwent a salvage RAMIE, the DFS was calculated from the start of dCRT to the day recurrent disease was diagnosed definitively by histopathologic assessment. Statistical analysis was performed using SPSS version 25 (SPSS, Chicago, IL, USA).

## Results

### Study Population

**Fig. 1:**
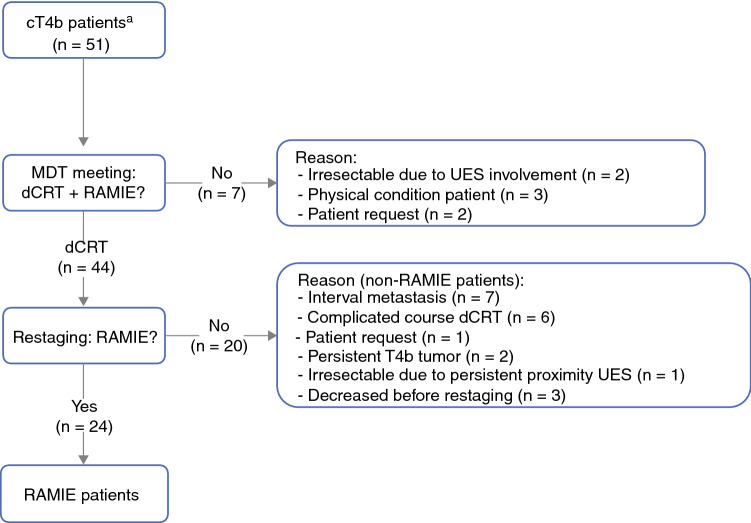
Flowchart of cT4b patients with an indication for surgical resection with curative intent through Robot Assisted Minimally Invasive Esophagectomy (RAMIE). *dCRT* definitive chemoradiotherapy, *MDT* multi-disciplinary team, *RAMIE* robot assisted minimally invasive esophagectomy, *UES* upper esophageal sphincter, ^a^ observed on PET-CT, EBUS and/or EUS

Between June 2012 and November 2019, 51 consecutive patients with cT4b esophageal cancer were presented at a multidisciplinary team meeting. Treatment with curative intent consisting of dCRT followed by restaging and salvage RAMIE was deemed possible for 44 of the patients. Of these 44 patients, 20 did not proceed to surgery (non-RAMIE patients) for reasons provided in the next section and in Fig. 1.

The patients who underwent a salvage RAMIE (RAMIE patients) and the non-RAMIE patients were comparable in terms of age (63 vs 65 years) and sex (79% vs 65% males). The predominant tumor characteristics in both groups were squamous cell carcinoma (96% vs 95%) with invasion of the tracheobronchial tree (67% vs 75%) and clinically suspected lymph nodes (92% vs 80%). The primary tumor in the RAMIE group was located mainly in the upper one-third of the esophagus, whereas the primary tumor in the non-RAMIE group was located in the middle one-third of the esophagus. In the RAMIE group, the median interval between completion of dCRT and surgery was 14 weeks (range, 6–21 weeks). A complete overview of the patient and tumor characteristics of both the RAMIE and non-RAMIE patients is presented in Table 1.

**Table 1:** Patient demographics and tumor characteristics of patients that proceeded to surgery (RAMIE) and patients in whom surgery was omitted (Non-RAMIE)

Characteristics	RAMIE	Non-RAMIE
	*n *=* 24*	*n *=* 20*
**Age, years** (mean ± SD)	63 ± 8	65 ± 8
*Sex*		
Male	19 (79%)	13 (65%)
Female	5 (21%)	7 (35%)
**BMI, kg/m**^**2**^ (mean ± SD)	23 ± 4	23 ± 4
*Co*-*morbidity*		
Vascular	11 (46%)	13 (65%)
Cardiac	2 (8%)	3 (15%)
Pulmonal	7 (30%)	1 (5%)
Diabetes	1 (4%)	2 (10%)
Oncologic	3 (13%)	3 (15%)
*ASA classification*		
1	2 (8%)	2 (10%)
2	14 (58%)	14 (70%)
3	8 (33%)	4 (20%)
*Histopathology*^a^		
Adenocarcinoma	1 (4%)	1 (5%)
Squamous cell carcinoma	23 (96%)	19 (95%)
*Site of tumor invasion*		
Tracheobronchial tree	16 (67%)	15 (75%)
Aorta	5 (21%)	2 (10%)
Combination	3 (13%)	3 (15%)
*Clinical N stage*^b^		
N0	2 (8%)	4 (20%)
N1	13 (54%)	9 (45%)
N2	7 (29%)	7 (35%)
N3	2 (8%)	0
*Tumor location*		
Upper 1/3	23 (96%)	9 (45%)
Middle 1/3	1 (4%)	11 (55%)

*BMI* Body Mass Index, *ASA* American Society of Anasthesiologists

^a^ Determined in pre-treatment biopsy

^b^ Based on the 7th edition of the American Joint Committee in Cancer (AJCC)

### Reasons Non-RAMIE Patients Did Not Proceed to Surgery

The 20 non-RAMIE patients did not proceed to surgery predominantly because of interval metastasis (*n* = 7) and complications during dCRT (*n* = 9). The complications during dCRT consisted mainly of fistulas to adjacent structures such as the lungs, bronchus, aorta, spine, and carotid artery. These complications resulted in death before surgery in three cases. Other reasons for not proceeding to surgery were persistent tumor invasion of adjacent structures during restaging (*n* = 2), persistent proximity of the primary tumor to the upper esophageal sphincter during restaging (*n* = 1), and patient request (*n* = 1).

### Clinical Staging and Restaging of RAMIE Patients

During clinical staging, involvement of the tracheobronchial tree was observed in 16 patients (67%), and involvement of the aorta was observed in 5 patients (21%). Both the tracheobronchial tree and the aorta were involved in three patients (13%). During restaging, a PET-CT was acquired in 22 patients.

A metabolic complete response of the primary tumor was observed in 6 patients (25%) and a partial metabolic response in 16 (67%) patients. In 18 patients, a re-staging EBUS was performed. In 15 patients, the initial invasion of the primary tumor into the tracheobronchial tree had dissipated, and normal anatomy was restored. Persistent ingrowth was suspected in three patients, but because certain differentiation between fibrosis and tumor was not possible, surgical exploration was performed. One of these patients eventually underwent an incomplete resection due to persistent tumor invasion into the tracheobronchial tree.

### Intraoperative Results of RAMIE Patients

In all patients, a reconstruction with a gastric conduit and an intrathoracic or cervical anastomosis was performed. The median duration of the RAMIE was 394 min (range, 319–684 min). The duration of the thoracoscopic phase was 151 min (range, 87–263 min). No conversions or major intraoperative complications occurred. The median blood loss was 250 ml (range, 60–1000 ml) (Table 2).

**Table 2:** Intraoperative and pathological data (n = 24)

Operating time, min - median (range)	394 (319–684*)
Thoracoscopic phase, min - median (range)	151 (87–263)
**Total blood loss, ml - median(range)**	250 (60-1000)
**Conversion (thorax and abdomen)**	0
**Intraoperative complications**	0
**Type of reconstruction**	
Gastric conduit (cervical, hand sewn, end-to-side)	23 (96)
Gastric conduit (intrathoracic, hand sewn, end-to-side)	1 (4)
**Radicality of procedure**	
R0	22 (92)
R1	2 (8)
**ypT-stage**	
T0N0	11 (46)
T0N1	2 (8)
T1N0	1 (4)
T2N0	5 (21)
T3N1	3 (13)
T3N2	1 (4)
T4aN1	1 (4)
**Mandard score**	
1	13 (54)
2	4 (17)
3	4 (17)
4	2 (8)
5	1 (4)
**Lymph node yield - median (range)**	27 (16–74)
**Metastatic lymph nodes - median (range)**	0 (0-4)

*210 min delay due to camera disfunction

### Postoperative Results of RAMIE Patients

An uncomplicated postoperative course was observed in two patients patients (8%). Complications with a Clavien Dindo grade 2 score or higher occurred in 20 patients (83%). Pulmonary complications were the most common. Pneumonia according to the Universal Pneumonia Score was observed in 11 patients (46%), requiring antibiotic treatment.^32^ Anastomotic leakage occurred in six patients (25%), all with mediastinal manifestation necessitating bedside or surgical drainage. In two of these patients, video-assisted thoracoscopic surgery (VATS) for pleural empyema was performed. The median intensive care unit (ICU) stay was 1 day (range, 0–17 days), and the median overall hospital stay was 14 days (range, 7–58 days).

In-hospital mortality occurred in one patient (5%). The cause of mortality was sepsis after anastomotic leakage with mediastinitis, resulting in multi-organ failure. The patient died after refraining from further treatment. A complete overview of the postoperative complications and hospitalization course is provided in Table 3.

**Table 3:** Postoperative complications and hospitalization course (n = 24)

Complication groups*	n (%)
**Uncomplicated procedures**	2 (8)
**Gastrointestinal**	
Anastomotic leakage	
Type II (nonsurgical therapy)	2 (8)
Type III (surgical therapy)	4 (16)
*Clostridium difficile* infection	1 (4)
**Pulmonary**	
Pneumonia (UPS)	11 (46)
Pleural effusion requiring drainage	2 (8)
**Cardiac**	
Myocardial infarction	1 (4)
Atrial fibrillation	7 (29)
**Neurologic**	
Recurrent nerve injury	
Type Ia (unilateral transient injury)	6 (25)
**Other**	
Chyle leakage	
Type I (< 1L/day, dietary modifications)	2 (8)
**Hosptalization course**	
**ICU stay, days - median (range)**	1 (1-17)
**Hospital stay, days - median (range)**	14 (7–58)
**In-hospital mortality**	1 (4)

Complications defined according to Esophageal Complications Consensus Group (ECCG). *Complication group items include patients with more than 1 complication. UPS; Uniform Pneumonia Score; ICU Intensive Care Unit, VATS; Video Assisted Thoracoscopic Surgery

### Histopathology of RAMIE Patients

A local pathologic complete response (Mandard 1) was observed in 13 patients (54%). Locoregional lymph nodes containing metastasis were found in two of these patients. A radical resection was achieved in 22 patients (92%). In one of the two patients who underwent an incomplete resection, both the circumferential and proximal resection margins were tumor positive. In the other patient, the circumferential resection margin was tumor positive. The median number of retrieved lymph nodes was 27 (range, 16–74), and the median number of positive lymph nodes was 0 (range, 0–4) (Table 2).

### Survival Outcomes of RAMIE and Non-RAMIE Patients

**Fig. 2:**
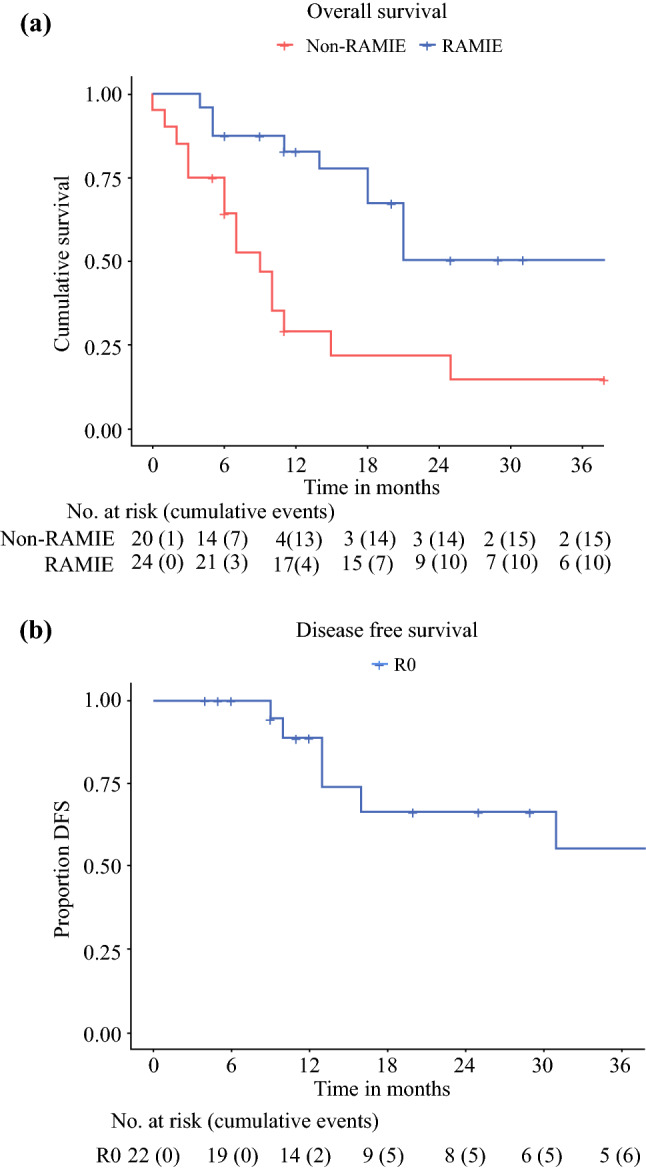
Kaplan Meier curves of the overall survival and disease free survival. **a** Overall survival of patients who underwent a salvage RAMIE (blue line) and patients who did not proceed to surgery after definitive chemoradiotherapy (red line), **b** Disease free survival of patients in whom a radical (R0) resection was achieved; RAMIE robot assisted minimally invasive esophagectomy

At the time of analysis, the median follow-up time for the entire group (RAMIE and non-RAMIE patients) was 11 months (range, 0–90 months). The overall survival rate for the non-RAMIE patients was 29% after 12 months and 21% after 24 months. For the RAMIE patients, the overall survival rate was 83% after 12 months and 51% after 24 months (Fig. 2A). The 22 patients in whom a radical resection was achieved had a cumulative DFS rate of 89% after 12 months and 68% after 24 months (Fig. 2B). Four of these patients (17%) experienced a local recurrence in combination with systemic metastasis. The locoregional recurrences were located at the site of the anastomosis in the gastric conduit (*n* = 2) or in the upper mediastinal lymph nodes (*n* = 2). Three patients (13%) experienced only systemic metastasis. At the time of analysis, 15 patients had no signs of local or distant recurrent disease.

## Discussion

This is the first study to assess the perioperative and oncologic outcomes of a salvage RAMIE after dCRT for patients with a cT4b esophageal carcinoma. The perioperative complication rates were acceptable. A radical resection was achieved in almost all patients, and a 24-month DFS rate of 68% was observed. These results demonstrate the feasibility of a curative salvage RAMIE for patients initially deemed inoperable.

In the current study, a radical resection rate of 92% (22 of 24 patients) was achieved. Recently, two phase 2 trials demonstrated that surgery is a feasible treatment option but that it should be reserved for patients in whom a radical resection can be achieved. In these trials, an open esophagectomy was performed, resulting in a radical resection rate of 39.6% (19 of 48 patients) in the one trial and 81.8% (9 of 11 patients) in the other trial.^17^,^19^

In randomized clinical trials, RAMIE after neoadjuvant chemoradiotherapy for resectable (cT1-4aNxM0) esophageal cancer has proved to be superior to open esophagectomy in terms of postoperative complications.^20^ Although no direct comparisons can be made for salvage esophagectomies, it could well be hypothesized that the surgical benefits accommodated by robotic assistance are even more prominent in downstaged (i.e.,fibrotic) and often upper esophageal (i.e., difficult to reach) T4b tumors.^22^,^23^ These benefits consist of a 3D enlarged view enabling dissection at a microscopic level and articulation of instruments allowing the surgeon to work precisely, even in the upper mediastinum. These advantages facilitate a radical resection without major intraoperative complications or conversions, as demonstrated in the current report.

The observed postoperative complications and in-hospital mortality rates indicate that higher risks are associated with a salvage RAMIE for cT4b tumors than with RAMIE for primary resectable esophageal cancer.^20^ The observed morbidity and mortality rates are comparable with those of previous reports on the surgical management of T4b esophageal cancer despite widely varying definitions and classifications of complications.^1^ The current report describes predominantly mild complications (Clavien Dindo grades 1 and 2) that were manageable without surgical reinterventions or ICU readmission. However, the observed increased risk of postoperative complications underscores the importance of thorough preoperative screening of patients considered for surgery after dCRT.

Pivotal in the selection of proper patients for a possible curative but highly invasive salvage RAMIE is assessment of the tumor’s resectability during restaging. The survival rates for cT4b patients in whom a radical resection is achieved are comparable to patients with resectable disease. Conventional diagnostic methods lack sufficient resolution for the visualization of anatomic boundaries between tumor and adjacent structures to assess resectability after dCRT. Imaging techniques such as EBUS and magnetic resonance imaging (MRI) provide a detailed overview of the anatomy of the esophagus and adjacent structures, enabling accurate assessment of resectability.^33^^–^35 Although promising, assessment of irradiated and fibrotic tissue remains challenging. Therefore, further research assessing the ability of EBUS to determine resectability is warranted and will provide valuable information on patient selection for a salvage RAMIE.

In the current study, a pathologic complete response of the primary tumor was observed in 13 patients (54%). This response rate is in line with previous literature on squamous cell carcinoma, indicating that selected patients might benefit from an organ-sparing approach after dCRT.^25^ During restaging, a complete metabolic response of the primary tumor on PET-CT was observed in six patients. Three of these patients had a pathologic complete response. The remaining pathologic complete responders exhibited a partial metabolic response during restaging. This demonstrates the inability of PET-CT to assess response accurately after neoadjuvant treatment. Therefore, the results of currently running trials in response assessment for resectable esophageal cancer should be awaited before omitting cT4b patients from surgery based on restaging with PET-CT.^36^ Because 54% of the patients had a complete response, decision-making in this respect should also include the functional outcome after intensive irradiation of the esophagus, which creates stenosis and scar formation, causing dysphagia with the need for repeated dilation and a subsequent decrease in the quality of life.^37^ Literature on patient-reported outcomes (e.g., quality of life) after dCRT in T4b tumors remains scarce, and more research is warranted because these factors should be taken into account when an organ-sparing approach is considered.^38^

Some limitations apply to the current study. A direct comparison between dCRT followed by surgery and dCRT alone could not be made. The non-RAMIE patients did not proceed to surgery due to factors that considerably affect prognosis such as complications from dCRT and progressive disease. A comparative analysis between the RAMIE and non-RAMIE patients would have been biased and was therefore not performed. However, the results of dCRT with comparable chemoradiotherapy regimens have been published, enabling an indirect comparison.

Reported locoregional failure rates reaching 50% after dCRT instead of the current 17% together with the observed survival rates demonstrate the possible advantages of a salvage RAMIE for T4b patients.^2^^–^7 Future comparative trials are warranted to validate these findings. Furthermore, the limited number of patients included in the current study can be considered as a limitation, increasing the susceptibility of the results to chance. Nevertheless, the current report describes one of the largest series of cT4b patients in the Western world. Therefore, these results contribute to the best available evidence and should act as an incentive to further assess the benefits from surgical treatment options after chemoradiotherapy for this patient population.

Finally, the high incidence of upper mediastinal squamous cell carcinomas in the current report is not representative of the general Western esophageal cancer population. This is most probably related to the predominant location of esophageal squamous cell carcinomas in the upper mediastinum, causing ingrowth in the tracheobronchial tree, aorta, or both at this level.

The current study aimed to assess the perioperative and oncologic outcomes of a salvage RAMIE after dCRT in T4b esophageal cancer. A radical resection rate of 92% was achieved, with a grade 2 or higher complication rate of 83%. Radical resection resulted in DFS rates of 89% at 12 months and 68% at 24 months. These results demonstrate the feasibility of a curative surgical approach for patients with initial inoperable esophageal cancer but underscore the importance of a proper preoperative patient selection.
